# Supplementary material for: OprF Impacts Pseudomonas aeruginosa Biofilm Matrix eDNA Levels in a Nutrient-Dependent Manner
Source: J Bacteriol. 2023 Jun 13;205(7):e00080-23. doi: 10.1128/jb.00080-23 (PMC10367591; doi:10.1128/jb.00080-23)
Supplement: Supplemental file 1 — Supplemental methods, Tables S1 to S6, and Fig. S1 to S8. Download jb.00080-23-s0001.pdf, PDF file, 10.4 MB [file jb.00080-23-s0001.pdf]

OprF impacts *Pseudomonas aeruginosa* biofilm matrix eDNA levels in a nutrient-dependent manner

Erin K. Cassin, Sophia A. Araujo-Hernandez, Dena S. Baughn, Melissa C. Londono, Daniela Q. Rodriguez, Natalie S. Al-Otaibi, Aude Picard, Julien R. C. Bergeron, and Boo Shan Tseng

SUPPLEMENTAL MATERIAL

Supplemental Methods
Supplemental Tables S1 – S7
Supplemental Figures S1 – S8
Supplemental References

SUPPLEMENTAL METHODS

Media, antibiotics, and antibodies

Bacteria were grown in liquid TSB (17 g/L tryptone, 3 g/L soytone, 5 g/L NaCl, 2.5 g/L glucose, 2.5 g/L K_2HPO_4), TSB 10 g/L NaCl (17 g/L tryptone, 3 g/L soytone, 10 g/L NaCl, 2.5 g/L glucose, 2.5 g/L K_2HPO_4), TSB no glucose (17 g/L tryptone, 3 g/L soytone, 5 g/L NaCl, 2.5 g/L K_2HPO_4), TSB no K_2HPO_4 (17 g/L tryptone, 3 g/L soytone, 5 g/L NaCl, 2.5 g/L glucose), LB (10 g/L tryptone, 5 g/L yeast extract, 10 g/L NaCl), LB 5 g/L NaCl (10 g/L tryptone, 5 g/L yeast extract, 5 g/L NaCl), LB w/ glucose (10 g/L tryptone, 5 g/L yeast extract, 10 g/L NaCl, 2.5 g/L glucose), and LB w/ K_2HPO_4 (10 g/L tryptone, 5 g/L yeast extract, 10 g/L NaCl, 2.5 g/L K_2HPO_4). For experiments involving induction of the P_{BADara} promoter, 0.5% L-arabinose (Sigma Aldrich) was added to the desired liquid medium. NSLB + 10% sucrose (10 g/L tryptone, 5 g/L yeast extract, 10% sucrose) was used during strain construction. Semisolid media contained 1.5% Bacto agar in LB or 1.0% Noble agar in VBMM (1). Antibiotics were used for strain construction only, at the following concentrations: for *E. coli*, 10 mg/L gentamicin, 50 mg/L kanamycin, 100 mg/L ampicillin; for *P. aeruginosa*, 60 mg/L gentamicin. Antibodies against OprF (CusaBio, CSB-PA318417LA01EZX) were used at 1/1000; and ecotin (2) at 1/400.

Construction of strains

P. aeruginosa strain sequences were acquired from the Pseudomonas Genome Database (www.pseudomonas.com)(3). Bacterial genomic DNA was obtained using the DNeasy Blood & Tissue Kit (Qiagen), and PCR fragments were purified using the Wizard SV Gel and PCR Clean-up System (Promega). Allelic exchange was used for gene deletions with the corresponding plasmid containing the deletion allele, as previously described (4). For the strain containing a miniTn7 construct, the allele was chromosomally integrated, as previously described (5, 6).

Construction of deletion alleles was performed using the regions flanking the gene of interest, produced by PCR of PAO1 gDNA using the respective UpF/UpR and DownF/DownR primer pairs. These fragments were then connected by SOE PCR and inserted into pDONRPEX18Gm using GateWay cloning (Invitrogen) to produce deletion vectors, which were confirmed via Sanger sequencing.

A miniTn7-based plasmid with *oprF* under the control of an arabinose-inducible promoter was made by first inserting *oprF* into a GateWay compatible vector using the PCR product of OEC01/03 with PAO1 gDNA. An LR reaction with this vector, pJJH187, and pUC18-miniTn7T2-Gm-GW was performed, resulting in a pDEST construct (pEC17).

Analysis of whole genome sequencing

Genomes of *P. aeruginosa* were sequenced using an Illumina NextSeq platform and sequencing reads were aligned to the appropriate PAO1 reference genome (3) at the Microbial Genome Sequencing Center (Pittsburgh, PA).

Growth rate assays

Cells from stationary phase cultures were seeded into flat-bottom polystyrene 96-well plates at an OD_{600} of 0.001. Absorbance at OD_{600} was read in a Synergy Hybrid HTX Microplate Reader (Bio-Tek Instruments) every 5 min for 16 h at 37˚C with shaking. The average of three biological replicates, which is the average of 4 technical replicates per strain, was reported. Growth rates were calculated by determining the slope of the growth curve in exponential phase.

Transmission electron microscopy of *P. aeruginosa*

Cell pellets were harvested by centrifugation at 8000 g at 4°C for 15 min. Cells were incubated overnight at 4°C in a premixed fixative solution of formaldehyde-glutaraldehyde (2.5% each) in 0.1 M sodium cacodylate buffer at pH 7.4 (Electron Microscopy Sciences). Cells were subsequently washed with a solution containing 1.2% sucrose and 4 mM CaCl2 in 0.1 M sodium cacodylate buffer, then subjected to a secondary fixation for 1 h at room temperature with 2% OsO4 (prepared in the above solution). After another wash, cells were stained for 1 h at room temperature with UranyLess, a solution containing lanthanides, and an alternative to uranyl acetate (Electron Microscopy Sciences). Cells were serially dehydrated with the following ethanol solutions (10 min each): 50%, 70%, 95% (two times), 100% (three times). Cells were gradually infiltrated with epoxy-type resin (EMbed 812 kit with BDMA, Electron Microscopy Sciences) through the following steps: ethanol was exchanged with 100% acetone (three times 10 min), then cells were exposed to a 1:1 acetone-resin mix for 1 hr at room temperature, to a 1:3 acetone-resin mix overnight, and to 100% resin (three times for 1hr). After a final exchange with fresh resin, samples were cured for 48 hr at 60°C. Ultrathin sections (70 nm) were cut using a Leica EM UC7 ultramicrotome, recovered on 200-mesh Cu grids, and double-stained with uranyl acetate and lead citrate for 10 and 4 min, respectively. Transmission electron microscopy was performed at 120 KeV on a JEOL TEM 1400Plus at the Electron Microscopy Core Laboratory of the University of Utah. Images were acquired with a Gatan Orius TM SC-1000 CCD camera (1 sec acquisition time).

For negative stain grid preparation and imaging, 5 µl of WT or Δ*oprF* culture grown in TSB or LB and grown to static or exponential phase was applied onto glow-discharged, carbon-coated copper grids (Agar Scientific). After incubating the sample for 2 min at room temperature, grids were rapidly washed in one drop of DI water and subsequently exposed to two successive short drops and one long incubation (30 sec) drop of UranyLess stain solution (Electron Microscopy Sciences). Images were recorded on a JEM1400 TEM (JEOL) equipped with a Matataki flash (JEOL) at 5000x and 12000x magnifications for all conditions.

Medium osmolarity

Medium osmolarity for 8 base media and variants (TSB, TSB 10g/L NaCl, TSB no glucose, TSB no K_2HPO_4, LB, LB 5 g/L NaCl, LB w/ glucose, LB w/ K_2HPO_4) was measured using a Vapro 5520 vapor pressure osmometer (Wescor). 10 µL of each medium was loaded onto sample discs (Wescor SS-033) and subjected to a 73 sec vaporization. The average of 3 readings per medium was reported.

Medium metal concentrations by ICP-MS

Each medium (TSB, TSB 10g/L NaCl, TSB no glucose, TSB no K_2HPO_4, LB, LB 5 g/L NaCl, LB w/ glucose, LB w/ K_2HPO_4) was filtered through a 0.22 μm nylon syringe filter (Rephile RephiQuik). Samples were diluted 1:100 in 2% nitric acid and injected into a ThermoFisher iCAP RQ inductively coupled plasma mass spectrometer.

Planktonic cell staining with crystal violet

Overnight culture was diluted 1:100 in fresh TSB and grown to mid-log growth phase (0.5 OD_{600}). 10-fold serial dilutions of mid-log cultures were spread on LB agar and incubated overnight for verification of cell counts (CFU/mL). Mid-log culture volumes corresponding to $5.0x10^7$, $7.5x10^7$, $1x10^8$, $1.25x10^8$, $1.5x10^8$, $2.0x10^8$ CFU/mL were centrifuged at 6000g for 8 min. Supernatant was removed by pipetting, and cell pellets were stained with 0.1% crystal violet, vortexed, and incubated for 15 min. Stained cells were centrifuged at 6000g for 8 min and were subsequently washed with 1mL deionized water three times (with centrifugation between washes). Stained biomass was eluted using 30% acetic acid, transferred to a flat-bottom 96-well plate (Greiner Bio-One, #655090), and the absorbance at OD_{550} was read in a Synergy Hybrid HTX Microplate Reader (BioTek Instruments). Absorbance from stained blank media tubes was subtracted from raw OD_{550} readings. Three biological replicates were averaged.

SUPPLEMENTAL TABLES

Supplemental Table S1. Media components of tryptic soy broth (TSB) and lysogeny broth (LB).

TSB		LB	
17 g/L	Tryptone	10 g/L	Tryptone
3 g/L	Soytone	5 g/L	Yeast extract
5 g/L	NaCl	10 g/L	NaCl
2.5 g/L	Glucose		
2.5 g/L	K_2HPO_4		

Supplemental Table S2. Statistical differences in static biofilm biomass grown in TSB versus LB.

	Strain	WT		ΔpslD		ΔoprF		ΔoprF + oprF		ΔoprF + oprF + ara	
Strain	Media	TSB	LB	TSB	LB	TSB	LB	TSB	LB	TSB	LB
WT	TSB			*		*		*			
	LB				*						
ΔpslD	TSB							*		*	
	LB						*		*		*
ΔoprF	TSB						*				
	LB										
ΔoprF + oprF	TSB										
	LB										
ΔoprF + oprF + ara	TSB										
	LB										

Significance was determined via a two-way ANOVA with post-hoc Bonferroni. Asterisk, $p < 0.05$. Open box, no statistical difference.

Supplemental Table S3. Statistical differences in static biofilm biomass grown in TSB and LB variants.

**TSB NaCl**	Strain	WT		ΔpslD		ΔoprF		ΔoprF + oprF		ΔoprF + oprF + ara	
Strain	NaCl	5	10	5	10	5	10	5	10	5	10
WT	5			*		*		*			
	10				*						*
ΔpslD	5							*		*	
	10						*		*		*
ΔoprF	5						*				
	10										
ΔoprF + oprF	5								*		
	10										*
ΔoprF + oprF + ara	5										
	10										

**LB NaCl**	Strain	WT		ΔpslD		ΔoprF		ΔoprF + oprF		ΔoprF + oprF + ara	
Strain	NaCl	5	10	5	10	5	10	5	10	5	10
WT	5			*		*		*			
	10				*						
ΔpslD	5					*		*		*	
	10						*		*		*
ΔoprF	5						*			*	
	10										
ΔoprF + oprF	5								*	*	
	10										
ΔoprF + oprF + ara	5										
	10										

**TSB glucose**	Strain	WT		ΔpslD		ΔoprF		ΔoprF + oprF		ΔoprF + oprF + ara	
Strain	Glucose	2.5	0	2.5	0	2.5	0	2.5	0	2.5	0
WT	2.5			*		*		*			
	0				*						*
ΔpslD	2.5							*		*	
	0						*		*		*
ΔoprF	2.5						*				
	0										
ΔoprF + oprF	2.5								*		
	0										
ΔoprF + oprF + ara	2.5										
	0										

**LB glucose**	Strain	WT		ΔpslD		ΔoprF		ΔoprF + oprF		ΔoprF + oprF + ara	
Strain	Glucose	0	2.5	0	2.5	0	2.5	0	2.5	0	2.5
WT	0			*							
	2.5				*		*		*		
ΔpslD	0					*		*		*	
	2.5						*		*		*
ΔoprF	0						*				
	2.5										
ΔoprF + oprF	0										
	2.5										*
ΔoprF + oprF + ara	0										
	2.5										

**TSB K_2HPO_4**	Strain	WT		ΔpslD		ΔoprF		ΔoprF + oprF		ΔoprF + oprF + ara	
Strain	K_2HPO_4	2.5	0	2.5	0	2.5	0	2.5	0	2.5	0
WT	2.5			*		*		*			
	0				*		*		*		*
ΔpslD	2.5							*		*	
	0						*		*		*
ΔoprF	2.5										
	0										*
ΔoprF + oprF	2.5								*		
	0										*
ΔoprF + oprF + ara	2.5										*
	0										

**LB K_2HPO_4**	Strain	WT		ΔpslD		ΔoprF		ΔoprF + oprF		ΔoprF + oprF + ara	
Strain	K_2HPO_4	0	2.5	0	2.5	0	2.5	0	2.5	0	2.5
WT	0			*							
	2.5				*						
ΔpslD	0					*		*		*	
	2.5						*		*		*
ΔoprF	0										
	2.5										
ΔoprF + oprF	0										
	2.5										
ΔoprF + oprF + ara	0										
	2.5										

Significance was determined via a two-way ANOVA with post-hoc Bonferroni. Asterisk, $p < 0.05$. Open box, no statistical difference.

Supplemental Table S4. Bacterial strains used in this study.

Strains	Relevant characteristics	Source
Escherichia coli		
NEB5α	For cloning; fhuA2 Δ(argF-lacZ)U169 phoA glnV44 Φ80Δ (lacZ)M15 gyrA96 recA1 relA1 endA1 thi-1 hsdR17	NEB
ccdB Survival2 T1R	F^- mcrA Δ(mrr-hsdRMS-mcrBC) Φ80lacZΔM15 ΔlacX74 recA1 araΔ139 Δ(ara-leu)7697 galU galK rpsL endA1 nupG fhuA::IS2, Sm^r	Invitrogen
S17.1 (λ_{pir})	For conjugation; F^- RP4-2-Tc::Mu *aphA*::Tn7 *recA* λ_{pir} lysogen, Sm^r, Tc^r	(7)
Pseudomonas aeruginosa		
PAO1	Wild-type PAO1	(8)
E2	Tomato plant isolate	(9)
BTPa1020	E2 containing an in-frame deletion of *oprF*	This study
MSH10	Water isolate	(9)
BTPa1021	MSH10 containing an in-frame deletion of *oprF*	This study
X24509	UTI isolate	(9)
BTPa1022	X24509 containing an in-frame deletion of *oprF*	This study
H103	PAO1 derivative; Cm^r auxotroph	(10)
H636	H103 *oprF*::Ω	(11)
PAO1 Δ*pslD*	PAO1 containing an in-frame deletion of *pslD*	(12)
BTPa768	PAO1 containing an in-frame deletion of *oprF*	This study
BTPa907	PAO1 Δ*oprF* *att*Tn7::P_{BADara}-*oprF*	This study
BTPa917	PAO1 containing an in-frame deletion of *pqsA*	This study
BTPa919	PAO1 containing an in-frame deletion of *pqsH*	This study
BTPa933	PAO1 containing in-frame deletions of *oprF* and *pqsA*	This study
BTPa935	PAO1 containing in-frame deletions of *oprF* and *pqsH*	This study

Supplemental Table S5. Plasmids used in this study.

Plasmids	Relevant characteristics	Source
pDONR221 P5-P2	GateWay-compatible vector with attP5 and attP2 recombination sites and *ccdB*; Kn^r, Cm^r	Invitrogen
pDONRPEX18Gm	pEX18-based, GateWay-compatible suicide vector (Accession No. KM880128) with *att*P1 and *att*P2 recombination sites and *ccdB*; Gm^r, Cm^r	(4)
pUC18-miniTn7T2-Gm-GW	GateWay-compatible miniTn7 vector with *att*R1 and *att*R2 recombination sites and *ccdB*; Ap^r, Gm^r, Cm^r	(12)
pDONRPUC18T-miniTn7T2-Gm	GateWay-compatible miniTn7 vector with *att*P1 and *att*P2 recombination sites and *ccdB*; Ap^r, Gm^r, Cm^r	(2)
pUCP22T2-GW	GateWay-compatible pUCP22 vector with attR1 and attR2 recombination sites and *ccdB*; Ap^r, Gm^r, Cm^r	(13)
pTNS2	T7 transposase expression vector; Ap^r	(5)
pFLP2	Plasmid that expresses Flp recombinase; Ap^r	(5)
pJJH187	pDONR221P1P5r-based Gateway-compatible vector encoding the *araC* repressor protein and the P_{araBAD} promoter; Kn^r	(14)
pEC17	pUC18-miniTn7::P_{BADara}-*oprF*	This study
pSA01	pENTR-pEX18-Δ*oprF*	This study
pDR10	pENTR-pEX18-Δ*pqsA*	This study
pDR11	pENTR-pEX18-Δ*pqsH*	This study

Supplemental Table S6. Oligonucleotides used in this study.

Primer	Sequence
OEC01 (*oprF* DownR)	ggggaccactttgtacaagaaagctgggtaTTACTTGGCTTCAGCTTCTACTTCG
OEC03 (*oprF* promoterless UpF)	ggggacaactttgtatacaaaagttgcgAGATGGGGATTTAACGGATGAAACTGAAG
OSA1 (*oprF* UpF)	CTCGAGGCTGGCGTAGGTG
OSA2 (*oprF* UpR)	ggggacaagtttgtacaaaaaagcaggctcaGATTGGATCGTTGGCTCGTCCATG
OSA3 (*oprF* DownF)	TAAGGTGTTCTTCAGTTTCATCCGTTAAATC
OSA4 (*oprF* DownR)	gatttaacggatgaaactgaagaacaccttaGAAGTAGAAGCTGAAGCCAAGTAATC
OSA5 (*oprF* SeqF)	ggggaccactttgtacaagaaagctgggtaGCTGCTGGCTGGAGGCATG
OSA6 (*oprF* SeqR)	GAGGAGGCTTCCGAGGAGAAG
OBT850 (*pqsA* UpF)	ggggacaagtttgtacaaaaaagcaggctcaATTTCAACAGGGAAGCCTGCAAATG
OBT851 (*pqsA* UpR)	GTTGGCCAATGTGGACATGACAGAAC
OBT852 (*pqsA* DownF)	gttctgtcatgtccacattggccaacGAGGAACGGGCATGTTGATTCAG
OBT853 (*pqsA* DownR)	ggggaccactttgtacaagaaagctgggtaCCATTCCTGCGCCGGATCAC
OBT854 (*pqsA* SeqF)	TATGGGGCATGTAGGTGTCCTCTTC
OBT855 (*pqsA* SeqR)	CCTACCTGGCGAATATCGGTATTC
OBT856 (*pqsH* UpF)	ggggacaagtttgtacaaaaaagcaggctcaGGAGCGGGGTCTGCGTATAG
OBT857 (*pqsH* UpR)	AACGGTCATCCGTTGCTCCTTAG
OBT858 (*pqsH* DownF)	ctaaggagcaacggatgaccgttGGTGAGATGGCCGCACAGTAG
OBT859 (*pqsH* DownR)	ggggaccactttgtacaagaaagctgggtaCATCTTCACGTCCATCAGGACGAC
OBT860 (*pqsH* SeqF)	GCTTCCGGCAATCACTACCTTG
OBT861 (*pqsH* SeqR)	GGTGACTACCACGACCTTGATGTC

Supplemental Table S7. Non-normalized static biofilm data from this study.
Provided separately as an Excel file.

SUPPLEMENTAL FIGURES

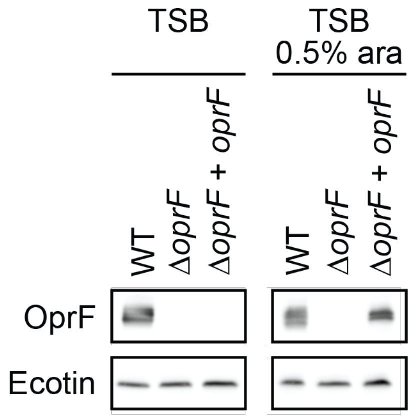

Supplemental Figure S1. OprF is expressed upon induction in the Δ*oprF* + *oprF* restoration strain.

OprF levels in planktonic PAO1 (WT), Δ*oprF*, and Δ*oprF att*Tn7::P_{BAD}-*oprF* (Δ*oprF* + *oprF* restoration strain) grown without (left) and with (right) 0.5% L-arabinose inducer (ara) were determined by Western blot. Ecotin was used as a loading control.

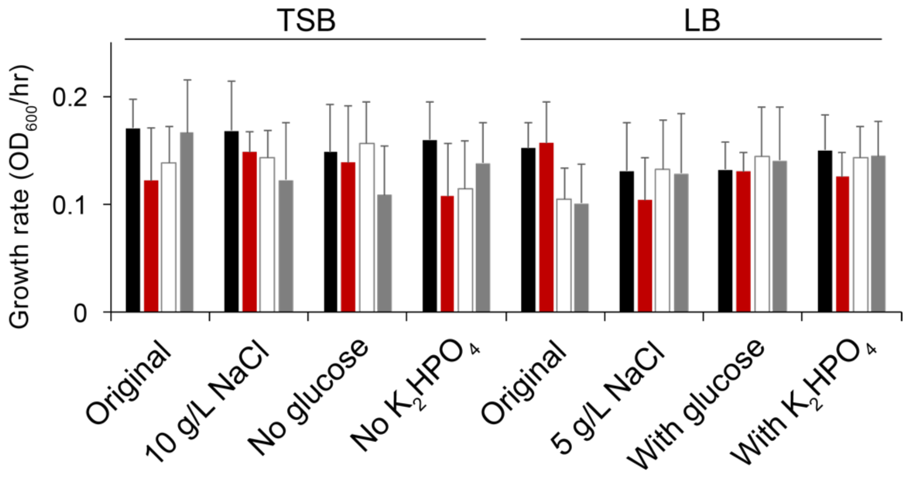

Supplemental Figure S2. No planktonic growth defects between strains in various media. Growth rate of PAO1 (WT, black), Δ*oprF* (red), and the Δ*oprF att*Tn7::P_{BAD}-*oprF* restoration strain (Δ*oprF* + *oprF)* without (white) and with (gray) 0.5% arabinose in the indicated media. Error bars, SEM (N = 3). No significant difference between strains within a medium or between media within a strain ($p > 0.05$; two-way ANOVA).

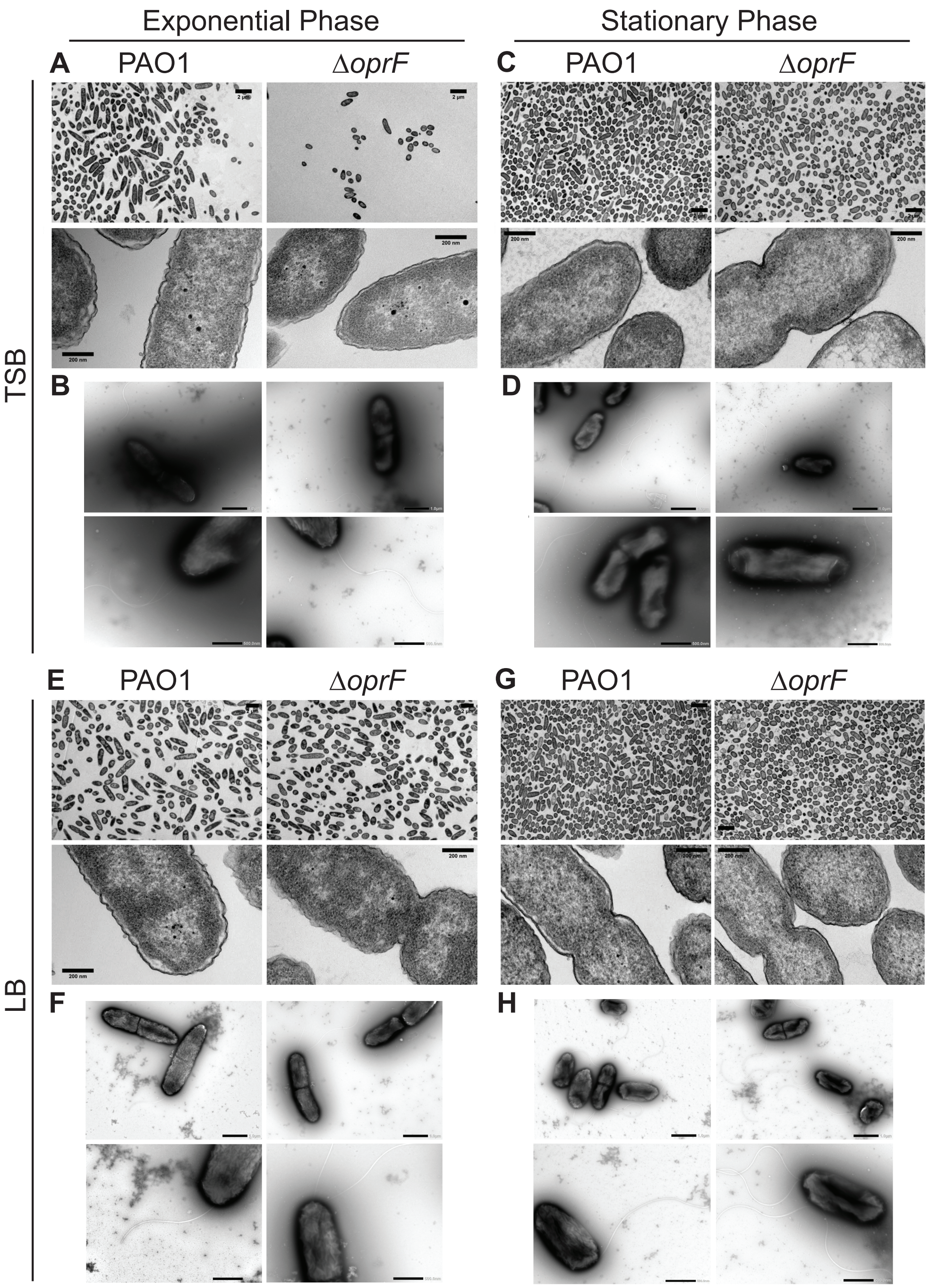

Supplemental Figure S3. No major cell morphology differences between wild type and Δ*oprF* grown in TSB or LB.

(A, C, E, G) Transmission electron micrographs (TEM) of PAO1 wild-type (left) and Δ*oprF* (right) cells grown in TSB (top row) and LB (bottom row) to exponential phase (0.6 OD_{600}; left column) and stationary phase (16 hr; right column). Note: the lower cell density of Δ*oprF* grown to exponential phase in TSB not representative of the whole pellet, which was homogeneous with other growth conditions). Bar, 2 µm (top) and 200 nm (bottom). (B, D, F, H) Negative-stained electron micrographs of samples as described above. Bar, 1 µm (top) and 500 nm (bottom).

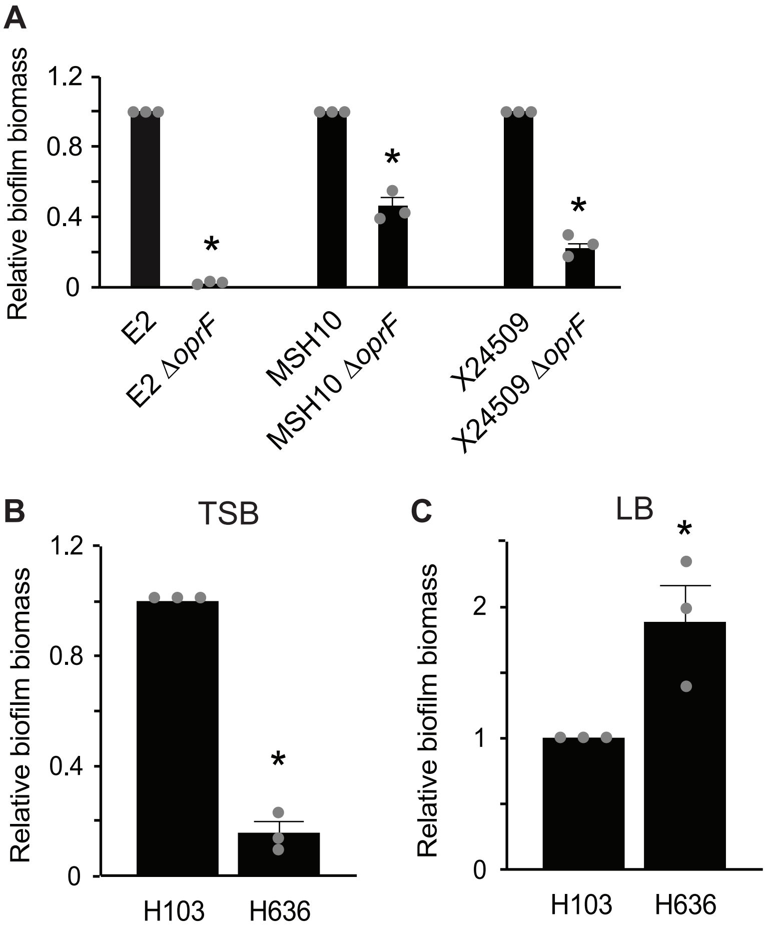

Supplemental Figure S4. Clinical and environmental Δ*oprF* strains form less biofilm in TSB.

(A) 24-hour static microtiter biofilm assays were performed with a tomato plant isolate (E2), a water isolate (MSH10), a UTI isolate (X24509), and their respective isogenic Δ*oprF* mutants in TSB. (B and C) 24-hour static microtiter biofilm assays were performed with an *oprF* interruption mutant (H636) and its parental strain (H103) in TSB and LB media. Biofilm formation was normalized to that of each respective parental strain grown in the same medium. Error bars, SEM (N = 3); dot, each biological replicate, which is the average of 3-6 technical replicates; asterisk, statistically different from the respective parental strain ($p < 0.01$; Student t-test).

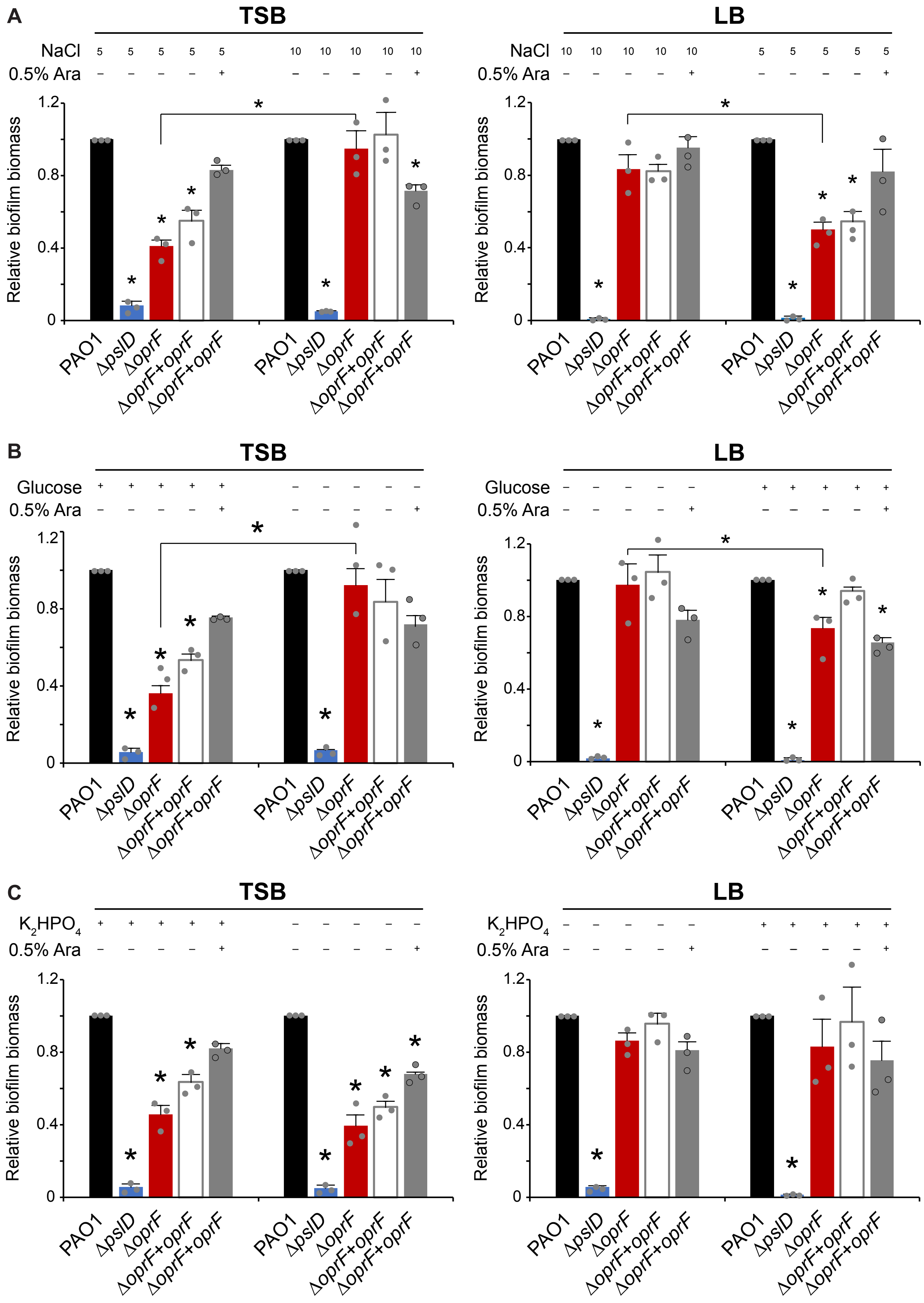
A
TSB
LB
NaCl
0.5% Ara
B
Glucose
C
K2HPO4
Relative biofilm biomass
PAO1
ΔpslD
ΔoprF
ΔoprF+oprF

Supplemental Figure S5. ∆*oprF* forms less biofilm in low sodium chloride and with glucose.

24-hour static microtiter biofilm assays were performed with *P. aeruginosa* PAO1 (WT, black), ∆*pslD* (blue), ∆*oprF* (red), and a ∆*oprF att*Tn7::P_{BAD}-*oprF* restoration strain (∆*oprF* + *oprF)* without (white) and with (gray) 0.5% arabinose (Ara) in the indicated media. Left column, TSB base medium and variants; right column, LB base medium and variants. (A) Biofilm formation in altered sodium chloride (NaCl); (B), glucose; and (C), dipotassium phosphate (K_2HPO_4) concentrations. Concentrations of altered medium component denoted in top row of each graph in g/L. Biofilm formation is normalized to WT in each respective medium. Error bars, SEM (N = 3); dot, each biological replicate, which is the average of 6 technical replicates; asterisk over bar, statistically different from WT in the same medium ($p < 0.05$; two-way ANOVA with post hoc Bonferroni). Statistical difference between ∆*oprF* strains in different media are indicated by a bar and asterisk. See Table S3 for full statistical comparisons.

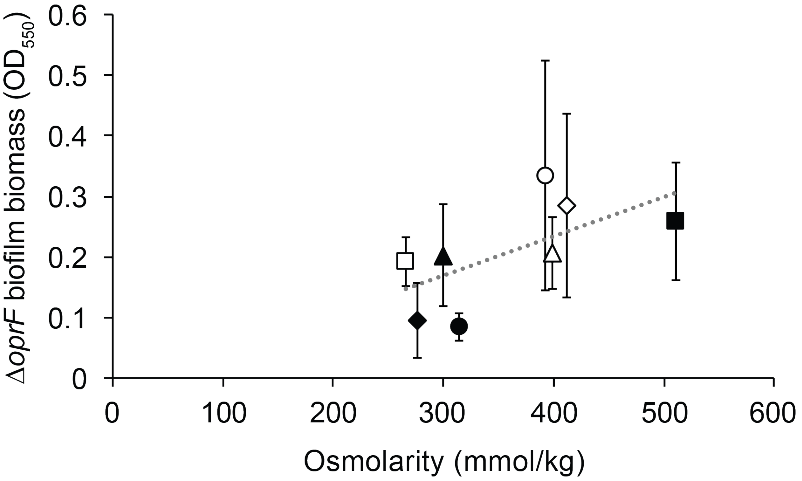

Supplemental Figure S6. Weak correlation that is not statistically significant between osmolarity and Δ*oprF* biofilm.

Osmolarity of each base medium and its variants compared to Δ*oprF* static microtiter biofilm biomass formed in respective medium. Osmolarity of original TSB base medium (black, circle), TSB 10 g/L NaCl variant (black, square), TSB with no glucose variant (black, triangle), TSB with no K_2HPO_4 variant (black, diamond), LB base medium (white, circle), LB 5 g/L NaCl variant (white, square), LB with 2.5 g/L glucose variant (white, triangle), and LB with 2.5 g/L K_2HPO_4 variant (white, diamond) were correlated to Δ*oprF* biofilm biomass in each respective medium (gray dotted line, $R^2 = 0.40541$; $p > 0.05$, Pearson's correlation).

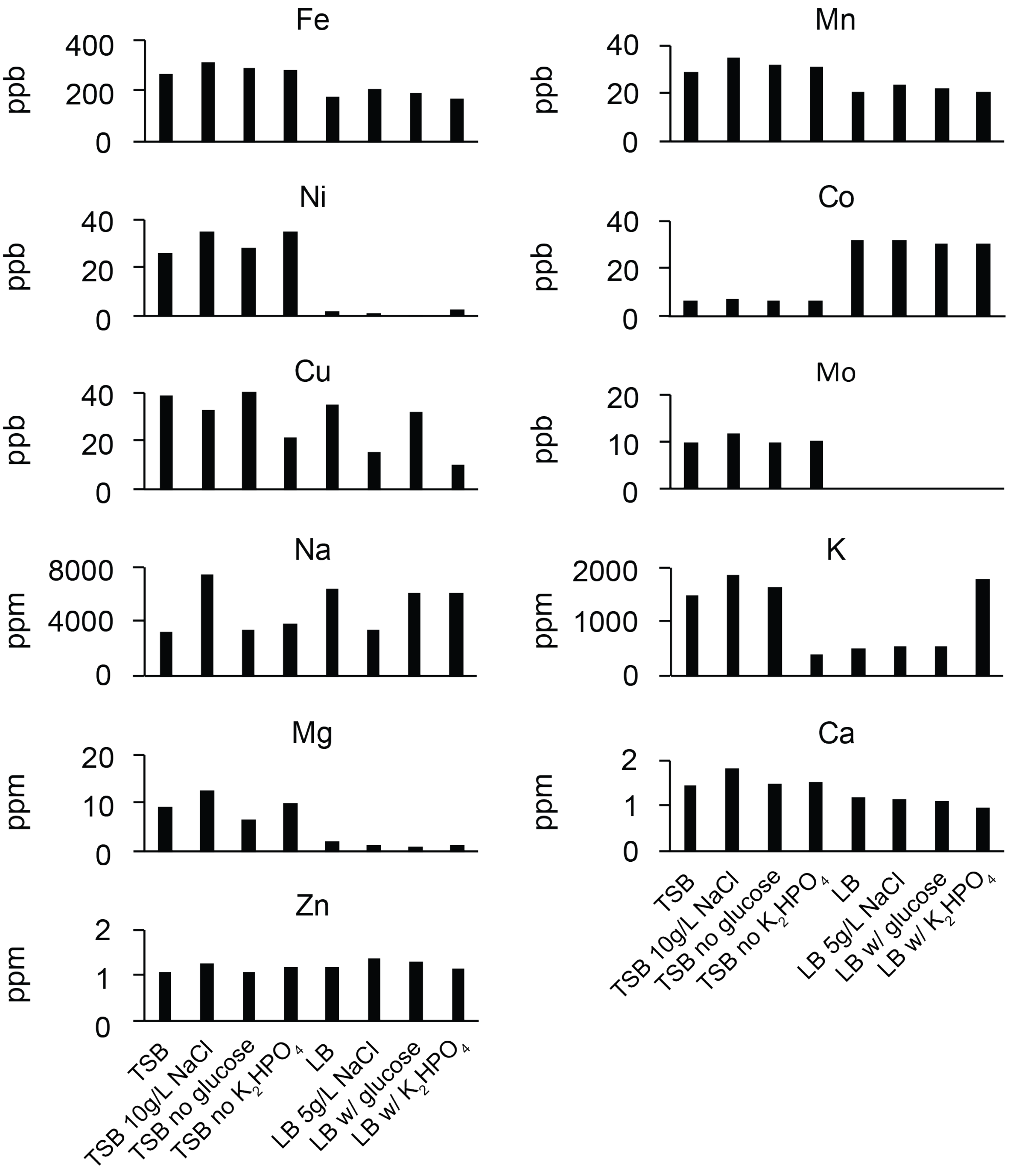

Supplemental Figure S7. Metal concentrations in various media.
Concentrations of biologically relevant metals (Fe, Mn, Ni, Co, Cu, Mo, Na, K, Mg, Ca, and Zn) in base media and variant formulations were determined by ICP-MS. Concentrations of each metal in parts per billion (ppb) or parts per million (ppm). No statistical significance between metal concentrations and Δ*oprF* biofilm formation ($p > 0.05$, Pearson's correlation).

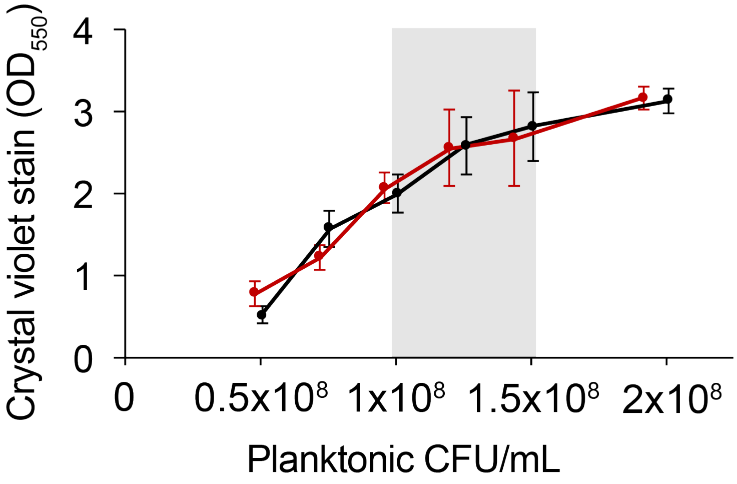

Supplemental Figure S8. Planktonic PAO1 and ∆*oprF* cells stain equivalently with crystal violet.

PAO1 (WT, black) and ∆*oprF* (red) cells grown in TSB at quantified cell densities (CFU/mL) and stained with 0.1% crystal violet exhibit equivalent absorbance. Gray box indicates the cell densities observed per well in biofilm cell viability assays in TSB. Error bars, SEM (N = 3).

SUPPLEMENTAL REFERENCES

1. Vogel HJ, Bonner DM. 1956. Acetylornithinase of Escherichia coli: partial purification and some properties. J Biol Chem 218:97-106.
2. Tseng BS, Reichhardt C, Merrihew GE, Araujo-Hernandez SA, Harrison JJ, MacCoss MJ, Parsek MR. 2018. A Biofilm Matrix-Associated Protease Inhibitor Protects Pseudomonas aeruginosa from Proteolytic Attack. MBio 9.
3. Winsor GL, Griffiths EJ, Lo R, Dhillon BK, Shay JA, Brinkman FS. 2016. Enhanced annotations and features for comparing thousands of Pseudomonas genomes in the Pseudomonas genome database. Nucleic Acids Res 44:D646-53.
4. Hmelo LR, Borlee BR, Almblad H, Love ME, Randall TE, Tseng BS, Lin C, Irie Y, Storek KM, Yang JJ, Siehnel RJ, Howell PL, Singh PK, Tolker-Nielsen T, Parsek MR, Schweizer HP, Harrison JJ. 2015. Precision-engineering the Pseudomonas aeruginosa genome with two-step allelic exchange. Nat Protoc 10:1820-41.
5. Choi KH, Schweizer HP. 2006. mini-Tn7 insertion in bacteria with single attTn7 sites: example Pseudomonas aeruginosa. Nat Protoc 1:153-61.
6. Hoang TT, Kutchma AJ, Becher A, Schweizer HP. 2000. Integration-proficient plasmids for Pseudomonas aeruginosa: site-specific integration and use for engineering of reporter and expression strains. Plasmid 43:59-72.
7. Kristensen CS, Eberl L, Sanchez-Romero JM, Givskov M, Molin S, De Lorenzo V. 1995. Site-specific deletions of chromosomally located DNA segments with the multimer resolution system of broad-host-range plasmid RP4. J Bacteriol 177:52-8.
8. Holloway BW. 1955. Genetic recombination in Pseudomonas aeruginosa. J Gen Microbiol 13:572-81.
9. Wolfgang MC, Kulasekara BR, Liang X, Boyd D, Wu K, Yang Q, Miyada CG, Lory S. 2003. Conservation of genome content and virulence determinants among clinical and environmental isolates of Pseudomonas aeruginosa. Proc Natl Acad Sci U S A 100:8484-9.
10. Hancock RE, Carey AM. 1979. Outer membrane of Pseudomonas aeruginosa: heat- 2-mercaptoethanol-modifiable proteins. J Bacteriol 140:902-10.
11. Woodruff WA, Hancock RE. 1988. Construction and characterization of Pseudomonas aeruginosa protein F-deficient mutants after in vitro and in vivo insertion mutagenesis of the cloned gene. J Bacteriol 170:2592-8.
12. Zhao K, Tseng BS, Beckerman B, Jin F, Gibiansky ML, Harrison JJ, Luijten E, Parsek MR, Wong GCL. 2013. Psl trails guide exploration and microcolony formation in Pseudomonas aeruginosa biofilms. Nature 497:388-391.
13. Almblad H, Harrison JJ, Rybtke M, Groizeleau J, Givskov M, Parsek MR, Tolker-Nielsen T. 2015. The Cyclic AMP-Vfr Signaling Pathway in Pseudomonas aeruginosa Is Inhibited by Cyclic Di-GMP. J Bacteriol 197:2190-200.
14. Khakimova M, Ahlgren HG, Harrison JJ, English AM, Nguyen D. 2013. The stringent response controls catalases in Pseudomonas aeruginosa and is required for hydrogen peroxide and antibiotic tolerance. J Bacteriol 195:2011-20.
